# Socioeconomic disparities in self-reported cardiovascular disease for Indigenous and non-Indigenous Australian adults: analysis of national survey data

**DOI:** 10.1186/1478-7954-8-31

**Published:** 2010-11-24

**Authors:** Joan Cunningham

**Affiliations:** 1 Menzies School of Health Research, Charles Darwin University, PO Box 41096, Casuarina, Northern Territory 0811, Australia

## Abstract

**Background:**

Little is known about the relationship between socioeconomic status (SES) and cardiovascular disease (CVD) among Indigenous Australians, or whether any such relationship is similar to that in non-Indigenous Australians.

**Methods:**

Weighted data on self-reported CVD and several SES measures were analyzed for 5,417 Indigenous and 15,432 non-Indigenous adults aged 18-64 years from two nationally representative surveys conducted in parallel by the Australian Bureau of Statistics in 2004-05.

**Results:**

After adjusting for age and sex, self-reported CVD prevalence was generally higher among those of lower SES in both the Indigenous and non-Indigenous populations. The relative odds of self-reported CVD were generally similar in the two populations. For example, the relative odds of self-reported CVD for those who did not complete Year 10 (versus those who did) was 1.4 (95% confidence interval [CI]: 1.1-1.8) among Indigenous people and 1.3 (95% CI: 1.2-1.5) among non-Indigenous people. However, Indigenous people generally had higher self-reported CVD levels than non-Indigenous people of the same age and SES group. Although smoking history varied by SES, smoking did not explain the observed relationships between SES and self-reported CVD.

**Conclusions:**

Socioeconomic disparities in self-reported CVD among Indigenous Australians appear similar in relative terms to those seen in non-Indigenous Australians, but absolute differences remain. As with other population groups, the socioeconomic heterogeneity of the Indigenous population must be considered in developing and implementing programs to promote health and prevent illness. In addition, factors that operate across the SES spectrum, such as racism, stress, dispossession, and grief, must also be addressed to reduce the burden of CVD.

## Background

Cardiovascular disease (CVD) is an important cause of morbidity and mortality, accounting for 32% of female deaths and 27% of male deaths worldwide in 2004 [1]. The CVD burden is particularly pronounced among Indigenous Australians, who are three times more likely than other Australians to die from CVD [2]. In 2001-05, CVD accounted for more than one-quarter of all excess Indigenous deaths [3].

In describing the global CVD burden, Yusuf and colleagues outlined five stages of the epidemiologic transition as they relate to CVD [4]. The Australian population overall is currently well advanced in its epidemiologic transition, with a preponderance of ischemic heart disease (IHD) and stroke occurring at relatively advanced ages (Stage 4). By contrast, Australia's Indigenous population is in an earlier stage of transition (Stage 3), characterized by earlier onset of IHD and stroke, as well as increasing prevalence of obesity and diabetes [4]. Rheumatic heart disease also remains prevalent among some parts of the Indigenous population [5], reflecting the ongoing infectious disease burden from earlier stages of the transition.

Socioeconomic status (SES) is associated with CVD, but the nature of the relationship has varied over time and place, depending in part on the stage of the CVD-related epidemiologic transition [4,6]. As has been the case in industrialized countries for the past several decades [6], low SES is associated with increased CVD morbidity and mortality among Australians overall [7,8].

Little is known about the relationship between SES and CVD within the Indigenous population. Previous reports have shown a significant relationship between low SES and increased prevalence of diabetes [9,10] and end-stage kidney disease [11,12], but not asthma [13].

The aim of the current study is to examine socioeconomic disparities in self-reported CVD among a nationally representative sample of Indigenous Australians, and to compare these with corresponding disparities in the non-Indigenous Australian population. It cannot be assumed that the relationships are the same in the two populations because specific measures of SES are not necessarily equivalent in different population groups; for example, they may have different meanings in different social groups, and they may not adequately measure all relevant aspects of what they purport to measure [14,15]. In addition, because different measures of SES are not necessarily interchangeable [14], a broad range of SES indicators is examined here.

## Methods

Data for Indigenous and non-Indigenous adults aged 18-64 years were taken from two national surveys conducted in parallel by the Australian Bureau of Statistics (ABS) in 2004-05: the National Aboriginal and Torres Strait Islander Health Survey (NATSIHS) and the National Health Survey (NHS). These two surveys had very similar content, and in most cases, the wording of questions on particular topics was identical [16]. This analysis is limited to responses to questions deemed by the ABS to be comparable between the two surveys [17].

Extensive details on survey methodology have been published elsewhere [16-21]. Briefly, both surveys were conducted using multistage sampling strategies. The first stage involved random selection of either communities or census collection districts, and subsequent stages involved selection of dwellings and individuals within households [18,21]. Both the NHS and NATSIHS samples were designed to provide reliable estimates for Australia as a whole as well as for selected subnational areas, such as state/territory, capital city versus balance of state within each state (NHS), and remote versus nonremote areas (NATSIHS). Indigenous respondents from the NHS were included with NATSIHS data to provide Indigenous population estimates [18]. Both surveys were limited to usual residents of private dwellings and conducted by trained ABS interviewers. Very remote areas were out of scope in the NHS but not the NATSIHS. In the NHS and in nonremote areas in the NATSIHS, data were collected using computer-assisted interviews. In remote areas of the NATSIHS, pen and paper interview forms were used, and some questions were simplified or deleted. The response rates in both surveys were relatively high. After accounting for sample loss (e.g., dwellings out of scope or vacant, households with no adults, etc.), 89% of selected households in the NHS were classified as fully/adequately responding [21]. Corresponding figures in the NATSIHS were 85.5% in remote Indigenous communities and 83.4% in other areas [18]. More details about the design, conduct, and results of the surveys are available elsewhere [16-21].

To allow interested researchers to access data, the ABS created a Confidentialized Unit Record File (CURF) for the NATSIHS. This file includes unit records for Indigenous respondents from the NATSIHS and the NHS, as well as unit records for non-Indigenous respondents from the NHS [17]. Although the CURF contains records for participants of all ages, this analysis is limited to 20,849 respondents (5,417 Indigenous and 15,432 non-Indigenous) aged 18-64 years. Those aged ≥65 years were excluded due to uncertainty about the applicability of socioeconomic indicators among older people, as well as the relatively small size of this group in the Indigenous population [3].

### Definition of CVD

Participants were classified as having CVD based on responses to several questions. Respondents were asked: 1) whether they had ever been told by a doctor or nurse that they had a heart or circulatory condition; 2) whether the condition(s) was long-term (lasting, or expected to last, ≥6 months); and 3) whether the condition was current. A prompt card shown or read to respondents included the following conditions: rheumatic heart disease; heart attack; stroke; angina; high blood pressure or hypertension; low blood pressure or hypotension; hardening of the arteries, atherosclerosis, or arteriosclerosis; fluid problems, fluid retention, or edema; high cholesterol; rapid or irregular heartbeats, tachycardia, or palpitations; heart murmur or heart valve disorder; hemorrhoids (nonremote areas only); varicose veins (nonremote areas only); and other (specify) [18].

For this analysis, self-reported CVD was defined as any self-reported current, long-term heart or circulatory condition(s). Although the CURF includes data on some of the individual conditions listed on the prompt card, the prevalence of most of these was too low (<5%) in the age range examined to warrant separate analysis. Also, because some conditions were not identified separately in the CURF, it was impossible to restrict CVD by excluding specific conditions, such as varicose veins and hemorrhoids, which were not included in the prompt list for respondents in remote areas.

### Socio-demographic factors

Information was available on a range of socioeconomic and demographic factors, including age, sex, educational attainment, nonschool qualifications, employment status, equivalized household income, home ownership (Indigenous respondents only), remoteness category, and area-level disadvantage, as well as smoking history (Table 1). Information about age, sex, and whether the respondent was currently attending school was provided by any responsible adult within the household. Information about the dwelling (including tenure) and the income of nonparticipant household members (required to calculate household income) was provided by a household spokesperson, chosen on the basis of his or her ability to provide accurate information. Information relating to geography (including remoteness classification and area-level disadvantage score) was provided by the ABS based on the census collection district in which the selected dwelling was located. All other information used in this analysis was provided by the respondent [18].

**Table 1:** Socio-demographic characteristics of Indigenous and non-Indigenous Australian adults aged 18-64 years, 2004-05*

	Indigenous % (95% CI)†	Non-Indigenous % (95% CI)†
Age (years)		
18-24	23.1 (21.7-24.4)	15.1 (14.8-15.4)
25-34	28.4 (27.7-29.0)	22.4 (22.3-22.6)
35-44	24.0 (23.5-24.5)	23.5 (23.4-23.7)
45-54	16.1 (15.7-16.4)	22.0 (21.8-22.1)
55-64	8.5 (7.1-9.9)	17.0 (16.9-17.1)
Male	46.8 (45.6-47.9)	49.8 (49.6-50.1)
Highest year of school completed		
Year 12	23.5 (21.2-25.8)	52.5 (51.2-53.8)
Year 11	13.0 (11.7-14.4)	10.9 (10.3-11.6)
Year 10	31.2 (29.4-33.1)	24.7 (23.7-25.7)
Year 9	13.9 (12.5-15.3)	6.3 (5.8-6.7)
Year 8 or less	17.3 (15.7-18.9)	5.4 (4.8-5.9)
Never went to school	1.1 (0.5-1.6)	0.2 (0.1-0.3)
Level of highest nonschool qualification		
Bachelor's degree or higher	4.8 (3.7-5.8)	20.8 (19.9-21.7)
Diploma	4.7 (3.7-5.7)	9.7 (9.1-10.3)
Certificate	24.2 (22.2-26.1)	26.0 (25.0-27.1)
No qualifications	66.4 (64.1-68.6)	43.5 (42.5-44.5)
Employment status		
Employed	54.7 (52.2-57.1)	76.1 (75.3-76.8)
Unemployed	8.1 (6.9-9.2)	3.0 (2.7-3.4)
Not in the labor force	37.3 (35.0-39.6)	20.9 (20.1-21.7)
Housing tenure		
Owner/purchaser	24.7 (22.1-27.3)	n/a‡
Renter or other tenure	75.3 (72.7-77.9)	n/a‡
Equivalized household income quintile§		
1 (lowest)	33.7 (31.4-36.1)	11.3 (10.7-11.9)
2	21.6 (19.7-23.6)	13.1 (12.5-13.8)
3	14.3 (12.4-16.1)	16.9 (16.1-17.6)
4	9.4 (7.7-11.2)	19.5 (18.7-20.2)
5 (highest)	5.2 (4.0-6.4)	21.7 (20.7-22.7)
Not known/not stated	15.6 (13.6-17.6)	17.5 (16.6-18.4)
SEIFA quintile||		
1 (most disadvantaged)	49.3 (43.7-55.0)	17.1 (15.7-18.5)
2	19.3 (15.2-23.3)	19.0 (17.4-20.7)
3	18.5 (14.3-22.7)	20.3 (18.4-22.2)
4	9.0 (6.4-11.6)	21.3 (19.5-23.0)
5 (least disadvantaged)	3.9 (2.2-5.7)	22.3 (20.0-24.7)
Remoteness category**		
Major cities	30.6 (29.1-32.0)	70.2 (68.6-71.8)
Inner regional	20.1 (19.0-21.3)	19.5 (17.9-21.0)
Outer regional	21.5 (20.4-22.5)	10.4 (9.2-11.5)
Remote or very remote	27.8 (26.3-29.4)	---
Smoking status		
Current smoker	53.5 (51.2-55.8)	25.7 (24.8-26.7)
Former smoker	16.8 (15.1-18.5)	23.2 (22.4-24.1)
Never smoker	29.7 (27.6-31.9)	51.0 (49.7-52.3)

* Source: Weighted data from the National Aboriginal and Torres Strait Islander Health Survey 2004-05 confidentialized unit record file (CURF) [17].

† CI, confidence interval. Proportions are weighted to provide population estimates. Totals are based on those with nonmissing data, except for equivalized household income, for which a separate category is shown.

‡ n/a, not available.

§ Gross weekly equivalized cash income of household, using the OECD scale [18]. Quintiles were determined based on all-Australian figures. That is, the same categories were used for both Indigenous and non-Indigenous respondents.

|| SEIFA, Socioeconomic Index for Areas, based on the 2001 Socioeconomic Indexes for Areas (SEIFA) Index of Disadvantage score for the census collection district of the selected dwelling [18]. Quintiles were determined based on all-Australian figures. That is, the same categories were used for both Indigenous and non-Indigenous respondents.

** Classified according to the Australian Standard Geographical Classification remoteness classification (based on the ARIA+ index) [18].

Those reported as still at school (n = 67) were not asked about educational attainment or nonschool qualifications. They were coded as missing on both variables. Those whose educational attainment was not stated (n = 2) and those whose level of qualifications could not be determined (n = 222) were coded as missing on this variable.

Gross weekly household equivalized income, which takes into account household size and composition, was estimated using the Organisation for Economic Co-operation and Development scale [18]. Quintiles were determined based on all-Australian figures. That is, the same categories were used for both Indigenous and non-Indigenous participants. Equivalized income quintile was not available for 2,941 respondents (14.1%). Analyses were conducted with these respondents coded as missing, as well as with them included using a special category of household income unknown.

Home ownership was only available in the CURF for Indigenous respondents (missing for n = 41), and was based on whether the home was owned or being purchased by any of its occupants (not necessarily the respondent) [18,20].

Remoteness category was classified according to the Australian Standard Geographical Classification remoteness classification (based on the ARIA+ index) into major cities, inner regional, outer regional, and remote/very remote [20]. ABS documentation indicates that the remote/very remote category was to be used for Indigenous respondents only [17]. Therefore, area of residence was recoded to missing for 312 non-Indigenous respondents whose residence was listed as remote/very remote.

Area-level disadvantage was based on the 2001 Socioeconomic Indexes for Areas (SEIFA) Index of Disadvantage score for the census collection district of the selected dwelling [18]. Quintiles were determined based on all-Australian figures. That is, the same categories were used for both Indigenous and non-Indigenous respondents. Those with SEIFA quintile not known (n = 313) were coded as missing.

Participants were also asked about their smoking history and were categorized as current, former, or never smokers.

### Statistical analysis

All analyses were conducted using Stata version 10.0 via the ABS's Remote Access Data Laboratory (RADL). Under the RADL system, analysts submit statistical code to the ABS; the code is then run and output made available through a password-protected Web account. Analysts do not have direct access to unit record data, and there are limits on commands and outputs that are allowed in order to protect the security and confidentiality of the data [22].

All analyses used ABS-generated person-weights (or expansion factors) to adjust for disproportionate sampling of some groups. The estimates produced in this manner apply to the population as a whole, and not just the sample [17,23]. Standard errors and 95% confidence intervals (CI) were calculated using replicate weights produced by ABS using the Jackknife method (250 replicate weights for Indigenous respondents, 60 for non-Indigenous respondents) [17,23]. These replicate weights allow estimation of standard errors, taking into account the complex design and weighting procedures used in the surveys [18,23]. Although Stata version 10 incorporates a suite of procedures to analyze complex survey data, these commands are not allowed in the RADL system (Therese Lalor, ABS, personal communication, May 2009). Instead, commands from the *svr *module written by Nick Winter (available using the Stata command: search svr, net) were used.

Directly age-standardized estimates and 95% CIs were calculated using an alternative set of person-weights and replicate weights produced by ABS for that purpose. The standard population was the total Australian population as of 30 June 2001 [17].

Logistic regression was conducted separately for Indigenous and non-Indigenous respondents due to different numbers of replicate weights for the two groups. All models were adjusted for age group and sex, with socioeconomic variables assessed individually and in combination. Household income was modeled using all five quintiles as well as comparing quintiles 1 and 2 with quintiles 3-5 combined, but only the latter results are presented. There were relatively few Indigenous participants in the top quintiles (Table 1), and preliminary analysis indicated a similar prevalence of self-reported CVD in the top three quintiles, allowing them to be combined with minimal loss of information. Participants with missing data were excluded only from analyses involving the variable for which they were missing data.

The potential role of smoking as a mediator of the relationship between SES and self-reported CVD was examined by adding smoking to models with age, sex, and SES variable(s), and assessing the change in the odds ratio for the SES variable(s).

### Ethics approval

This study was approved by the Human Research Ethics Committee of the Northern Territory Department of Health and Families and the Menzies School of Health Research. Both the Aboriginal subcommittee and the main committee considered and approved the proposal.

## Results

**Figure 1:**
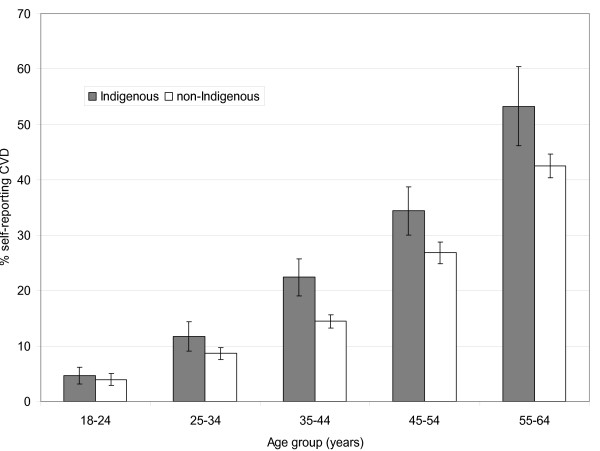
**Prevalence of self-reported CVD by age and Indigenous status, Australian adults, 2004-05**. Source: Weighted data from the National Aboriginal and Torres Strait Islander Health Survey 2004-05 confidentialized unit record file (CURF) [17].

Almost one in five Indigenous and non-Indigenous people aged 18-64 reported current, long-term CVD (Indigenous: 19.8%, 95% CI: 18.1-21.6; non-Indigenous: 19.1%, 95% CI: 18.4-19.8). Self-reported CVD increased with age among Indigenous and non-Indigenous people alike, but was more common among Indigenous people in all age groups (Figure 1).

The socio-demographic profile of the Indigenous population was significantly different from the non-Indigenous population (Table 1), with younger age distribution, lower educational attainment, and greater levels of disadvantage across a range of indicators.

**Figure 2:**
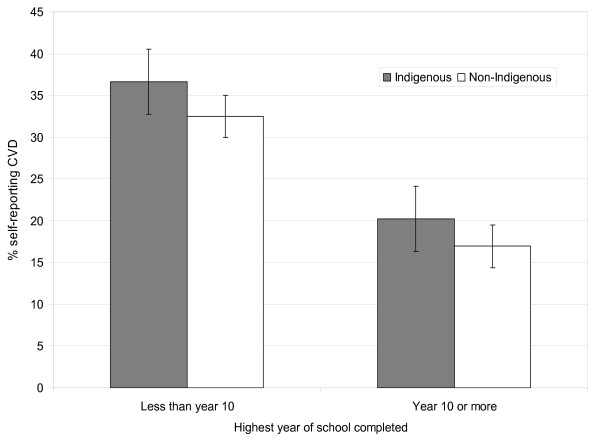
**Age-standardized prevalence of self-reported CVD by educational attainment for Indigenous and non-Indigenous Australian adults, 2004-05**. Source: Weighted data from the National Aboriginal and Torres Strait Islander Health Survey 2004-05 confidentialized unit record file (CURF) [17].

**Figure 3:**
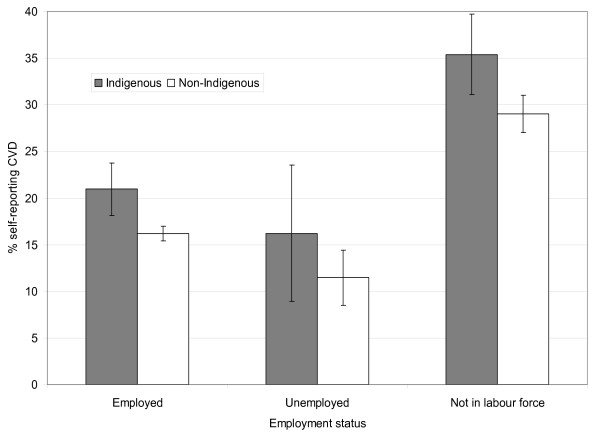
**Age-standardized prevalence of self-reported CVD by labor force status for Indigenous and non-Indigenous Australian adults, 2004-05**. Source: Weighted data from the National Aboriginal and Torres Strait Islander Health Survey 2004-05 confidentialized unit record file (CURF) [17].

Age-standardized self-reported CVD prevalence was generally higher for Indigenous people than non-Indigenous people of the same SES group (see, for example, Figures 2 and 3).

After adjusting for age and sex, several SES indicators were significantly associated with self-reported CVD in both groups, including education, employment status, household income, and remoteness category (Table 2). On each of these measures, the relative odds of self-reported CVD for those of lower SES were generally similar in the two populations. For example, the relative odds of self-reported CVD for those who did not complete Year 10 (compared with those who did) was 1.4 (95% CI: 1.1-1.8) among Indigenous people and 1.3 (95% CI: 1.2-1.5) among non-Indigenous people. Compared with people in the top three household income quintiles, the relative odds of self-reported CVD for those in the lowest quintile was 1.5 (95% CI: 1.1-2.0) among Indigenous people and 1.4 (95% CI: 1.2-1.6) among non-Indigenous people.

**Table 2:** Relative odds of self-reported cardiovascular disease* by socioeconomic status for Indigenous and non-Indigenous Australian adults aged 18-64 years, 2004-05†

	Indigenous		Non-Indigenous	
	**Adjusted for age and sex** **OR (95% CI)‡**	**Adjusted for age, sex and smoking** **OR (95% CI)‡**	**Adjusted for age and sex** **OR (95% CI)‡**	**Adjusted for age, sex and smoking** **OR (95% CI)‡**

Highest year of school completed				
Year 10 or more	1.0	1.0	1.0	1.0
<Year 10§	**1.4 (1.1-1.8)**	**1.4 (1.1-1.7)**	**1.3 (1.2-1.5)**	**1.4 (1.2-1.5)**
Has nonschool qualifications				
Yes	**0.8 (0.6-1.0)**	0.8 (0.6-1.0)	1.0 (0.9-1.1)	1.0 (0.9-1.1)
No	1.0	1.0	1.0	1.0
Employment status				
Employed	1.0	1.0	1.0	1.0
Unemployed	1.0 (0.6-1.7)	1.0 (0.6-1.6)	0.9 (0.6-1.2)	0.9 (0.7-1.2)
Not in labor force	**1.4 (1.1-1.8)**	**1.4 (1.1-1.8)**	**1.5 (1.3-1.7)**	**1.5 (1.3-1.7)**
Housing tenure				
Owner/purchaser	0.8 (0.6-1.0)	0.8 (0.6-1.1)	---	---
Renter/other tenure	1.0	1.0	---	---
Equivalized household income quintile||				
1 (lowest)	**1.5 (1.1-2.0)**	**1.5 (1.1-2.0)**	**1.4 (1.2-1.6)**	**1.4 (1.3-1.6)**
2	**1.8 (1.2-2.5)**	**1.7 (1.2-2.5)**	**1.4 (1.2-1.6)**	**1.4 (1.2-1.6)**
3-5 (highest)	1.0	1.0	1.0	1.0
Not known/not stated	1.3 (0.9-1.8)	1.2 (0.8-1.8)	**0.8 (0.7-1.0)**	**0.8 (0.7-1.0)**
SEIFA quintile**				
1 (most disadvantaged)	1.0 (0.5-2.0)	1.0 (0.5-1.9)	**1.6 (1.4-2.0)**	**1.7 (1.4-2.0)**
2	0.8 (0.4-1.7)	0.8 (0.4-1.7)	**1.5 (1.2-1.7)**	**1.5 (1.2-1.8)**
3	0.8 (0.4-1.8)	0.8 (0.4-1.7)	**1.3 (1.1-1.5)**	**1.3 (1.1-1.5)**
4	0.8 (0.4-1.8)	0.8 (0.4-1.8)	1.2 (1.0-1.4)	1.2 (1.0-1.4)
5 (least disadvantaged)	1.0	1.0	1.0	1.0
Remoteness category				
Major cities	1.0	1.0	1.0	1.0
Inner regional	1.3 (0.9-1.9)	1.3 (0.9-2.0)	**1.2 (1.0-1.3)**	**1.2 (1.0-1.3)**
Outer regional	1.0 (0.7-1.4)	1.0 (0.7-1.4)	0.9 (0.8-1.1)	0.9 (0.8-1.1)
Remote/very remote	**1.4 (1.0-1.9)**	**1.4 (1.0-1.9)**	---	---

*Includes: rheumatic heart disease; heart attack; stroke; angina; high blood pressure or hypertension; low blood pressure or hypotension; hardening of the arteries, atherosclerosis, or arteriosclerosis; fluid problems, fluid retention, or edema; high cholesterol; rapid or irregular heartbeats, tachycardia, or palpitations; heart murmur or heart valve disorder; hemorrhoids (nonremote areas only); varicose veins (nonremote areas only); and other relevant conditions as reported by participants [18].

†Source: Weighted data from the National Aboriginal and Torres Strait Islander Health Survey 2004-05 confidentialized unit record file (CURF) [17].

‡OR, odds ratio. CI, confidence interval.

§ Includes those who never went to school.

|| Gross weekly equivalized cash income of household, using the OECD scale [18]. Quintiles are based on national figures. That is, the same categories were used for both Indigenous and non-Indigenous respondents.

** SEIFA, Socioeconomic Index for Areas, Index of Relative Disadvantage. Quintiles are based on national figures. That is, the same categories were used for both Indigenous and non-Indigenous respondents.

Area level of disadvantage, as measured by SEIFA quintile, was significantly associated with self-reported CVD among non-Indigenous people but not among Indigenous people. Having a nonschool qualification was associated with lower self-reported CVD reporting among Indigenous people but not among non-Indigenous people.

### Smoking as a potential mediator

Current smoking was reported by 53.5% of Indigenous people and 25.7% of non-Indigenous people (Table 1). Smoking was significantly associated with most SES measures in both groups. For example, among Indigenous people, the prevalence of current smoking according to equivalized household income quintile was, from lowest to highest quintile: 62%, 58%, 45%, 39%, and 35% (*p *for trend <0.001). Corresponding non-Indigenous figures were: 35%, 31%, 25%, 25%, and 20% (*p *for trend <0.001).

Despite its social patterning, smoking did not explain the relationship between SES measures and self-reported CVD. Various adjustments for smoking did not materially change associations between SES variables and self-reported CVD among either group (Table 2). For example, the relative odds of self-reported CVD for Indigenous people who did not complete Year 10 (compared with those who did) was 1.4 (95% CI: 1.1-1.7) in a model adjusting for age groups, sex, and current and former smoking, compared with 1.4 (95% CI: 1.1-1.8) in a model adjusting for age groups and sex only.

## Discussion

The results presented here indicate that the apparent relationship between various SES measures and self-reported CVD is generally similar in relative terms for Indigenous and non-Indigenous Australians, despite these groups being in different stages of the epidemiologic transition with respect to CVD. However, SES did not explain all of the excess risk of self-reported CVD among Indigenous people, as age-adjusted self-reported CVD prevalence was generally higher for Indigenous people than for non-Indigenous people of the same SES level. Despite its marked social patterning in both populations, smoking did not explain the relationship between SES measures and self-reported CVD.

The combination of a socioeconomic gradient within the Indigenous population and a gap between Indigenous and non-Indigenous people of the same age and SES group suggests that traditional risk factors may not be sufficient to explain completely the patterns of self-reported CVD among Indigenous Australians. Other factors that may operate across the socioeconomic spectrum, including racism and discrimination [24-26], stress [27,28], and a legacy of grief, loss, and dispossession [29], may also play a role through a range of neuroendocrine, autonomic, metabolic, immune, and/or behavioral pathways [30]. A recent study indicated that racial disparities in diabetes prevalence in the US may be explained by differences in the "health risk" environments in which African Americans and whites live [31], and this could be relevant to CVD in Australia. Although genes clearly play a role in the development of CVD, the relationship between ethnicity and genetic susceptibility is quite complex [32,33].

The main strengths of this study are that it uses nationally representative data and identical measures of SES to make comparisons between Indigenous and non-Indigenous Australians. Although bias is always possible in any survey with less than complete participation, the relatively high response rates in both the NHS and the NATSIHS suggest that any such bias is unlikely to be large. The main limitations of the study relate to its cross-sectional nature, the potential misclassification of both CVD and SES, the heterogeneity of conditions included under CVD, and the exclusion of those most affected by CVD.

Because information on SES and self-reported CVD was collected at the same time, the temporal relationship between SES indicators and self-reported CVD is not always certain. For example, employment status may change as a consequence of chronic disease, which may explain the observed relationship between self-reported CVD and being out of the labor force.

Although most participants reporting CVD said they had been told by a health practitioner, it is possible some people who reported CVD did not actually have it, while others who did have CVD did not report it. Underreporting of CVD could have occurred as a result of lower access to or use of diagnostic services, limited health literacy, and/or higher thresholds for reporting relevant conditions that are not life-threatening or for which no treatment is required. If such factors were more common among those of lower SES, this could have resulted in an underestimation of the relationship between SES variables and CVD in both populations of interest. However, the extent of any such bias may not have been the same in the Indigenous and non-Indigenous populations.

Self-reported CVD included a broad range of conditions, from varicose veins and hemorrhoids to heart attack and stroke. This heterogeneity may have resulted in misclassification, which in turn may have affected the observed relationships. Because some conditions were not identified separately in the CURF, it was impossible to restrict self-reported CVD by excluding specific conditions, such as varicose veins and hemorrhoids, which were not included in the prompt list for respondents in remote areas. For many of the conditions that were identified separately, the prevalence among 18- to 64-year-olds was too low to warrant separate analysis.

Information used to determine SES may have been incorrectly reported by some participants, and only limited detail was available on the SES indicators examined. Despite the use of comparable scales, equivalence of a given level of SES may not be guaranteed across individuals or population groups [14,15]. For example, the meaning of a certain level of education may vary over time and place, and years of education do not necessarily reflect the quality of education received, nor its social or economic value [34,35]. Similarly, the use of SEIFA quintiles based on the whole population may not adequately capture the socioeconomic position of subgroups such as Indigenous Australians [36]. This may explain, at least in part, the lack of an apparent association between SEIFA quintile and self-reported CVD among Indigenous Australians. No information was available about other potentially important SES measures, such as childhood SES or household assets. Although an area-based measure of disadvantage was included, no other information was available about neighborhood/area characteristics. Equivalized household income is intended to adjust for household size and economies of scale, but the dynamic nature of Indigenous households [37] can make it difficult to assess household income and size, both of which are required to calculate equivalized income.

Although the NATSIHS and the NHS are both population-based, they are limited to the noninstitutionalized population. Those in hospitals and nursing homes at the time of the study were excluded, as were those not surviving long enough to participate. Thus, people with the most severe disease were likely to be excluded from both surveys. If this includes those most affected by smoking, this could explain, at least in part, the failure of smoking to explain the relationship between SES measures and self-reported CVD in this cross-sectional analysis; a longitudinal study might produce a different result. Although the prevalence of CVD increases with age, people aged 65 years and over were excluded from this analysis, and it is not clear whether the relationships observed among younger people would apply in older age groups.

Despite these limitations, the findings reported here are generally consistent with other studies of SES and cardiovascular-related outcomes. SES is strongly associated with cardiovascular disease incidence, prevalence, and mortality in developed countries and associated with most cardiovascular disease risk factors [6]. In a recent US study, SES (based on a combination of income and education) was significantly associated with estimated 10-year global CVD risk in all racial/ethnic groups except foreign-born Mexican American men. By contrast, the relationships between race/ethnicity and CVD risk *within *SES strata were inconsistent [38]. Although this might appear to suggest that SES may be more salient than race/ethnicity, other work indicates that both are important [15]. SES has also been associated with subclinical measures of CVD, such as carotid artery calcification and intima-media thickness, although these relationships have not been consistent across racial/ethnic groups [39]. SES has been associated with stroke incidence and mortality, as well as with stroke risk factors, although there is uncertainty about the extent to which these risk factors mediate the relationship between SES and stroke [40]. Similarly, SES appears to be associated with high blood pressure, although different measures of SES have been used in different studies, places, and populations [41].

Little is known about the relationship between SES and CVD among Indigenous Australians, but recent studies have examined the relationship between SES and other chronic diseases in this population. Cass and colleagues showed a strong gradient in regional rates of end-stage kidney disease (ESKD) according to an index of social disadvantage among Indigenous Australians [11]. Even in the least disadvantaged regions, the age- and sex-standardized ESKD incidence was generally significantly higher for Indigenous Australians than for the total Australian population [12]. Two recent studies of diabetes have shown marked gradients among Indigenous Australians according to a number of personal, household, and area measures of SES [9,10]. By contrast, no significant association was observed between traditional SES variables and asthma [13].

The observed relationship between SES and self-reported CVD in the current study is largely consistent with these earlier studies of kidney disease and diabetes, although the gradients are less steep than those previously seen for diabetes in the same group of NATSIHS/NHS participants [10]. For example, among Indigenous people who did not complete Year 10 (compared with those who did), the relative odds of diabetes was 1.8 (95% CI: 1.4-2.3); for self-reported CVD, it was 1.4 (1.1-1.8). Compared with Indigenous people in the top three household income quintiles, the relative odds of diabetes among those in the lowest income quintile was 2.3 (1.6-3.4), compared to 1.5 (1.1-2.0) for self-reported CVD [10]. However, the SES gradients were also less steep among non-Indigenous people: the odds ratio for <Year 10 was 1.8 (1.4-2.4) for diabetes, compared to 1.3 (1.2-1.5) for self-reported CVD; the odds ratio for lowest income quintile was 2.0 (1.5-2.8) for diabetes, compared to 1.4 (1.2-1.6) for self-reported CVD [10]. These differences may reflect the heterogeneity of conditions included as CVD and the various underlying SES gradients associated with them. They may also reflect differences in the quality of self-reporting of diabetes and the various components of CVD, real differences in their relationships with the SES variables considered, or a combination of these and other factors.

Despite tremendous diversity in areas such as language, culture, geography, and living conditions, Australia's Indigenous population is generally treated as a homogeneous entity. This is due in part to historical limitations in data quality and availability that inhibited finer analysis. However, recent developments, such as the implementation of Indigenous health and social surveys that run in parallel with corresponding mainstream collections mean that better data are now becoming available. It is imperative that public health researchers and policymakers take advantage of these data improvements and use the information to gain a more nuanced understanding of the Indigenous population. For example, Thomas and colleagues recently analyzed data on the social determinants of Indigenous nonsmoking and recommended that tobacco control programs consider additional targeting of more disadvantaged groups within the Indigenous population [42]. In addition, work is urgently needed to improve the measurement and analysis of SES among Indigenous Australians. This could include: 1) improving the measurement and meaningfulness of existing SES variables, such as household income; 2) collecting information on specific aspects of SES that are not currently available, such as childhood SES, household assets, and neighborhood amenities; and 3) developing a more suitable index that integrates several key SES measures.

## Conclusions

As with other population groups, socioeconomic heterogeneity in the Indigenous population must be considered in the development and implementation of programs to promote health and prevent illness. Although necessary and appropriate, such efforts may not completely eliminate the higher CVD burden among Indigenous Australians. While low SES is a significant risk factor for self-reported CVD among Indigenous Australians, and the magnitude of this relationship appears similar to that for other Australians, Indigenous Australians remain at higher absolute risk than their non-Indigenous peers of the same age and SES group. Factors that operate across the socioeconomic spectrum, such as racism, stress, dispossession, and grief, must also be addressed to reduce the excess burden of CVD in Indigenous Australians.

## Competing interests

The author declares that she has no competing interests.

## Authors' contributions

JC conceived of and designed the study, arranged for access to the data, performed all data analysis and interpretation, drafted and revised the manuscript, and gave final approval for submission.

## References

[B1] World Health Organization The Global Burden of Disease: 2004 Update 2008 Geneva: WHO Press

[B2] Australian Institute of Health and Welfare Cardiovascular disease and its associated risk factors in Aboriginal and Torres Strait Islander peoples 2004-05. Cardiovascular disease series no. 29. Cat. no. CVD 41 2008 Canberra: AIHW

[B3] Australian Bureau of Statistics & Australian Institute of Health and Welfare The Health and Welfare of Australia's Aboriginal and Torres Strait Islander Peoples 2008 Canberra: Australian Bureau of Statistics & Australian Institute of Health and Welfare

[B4] Yusuf S Reddy S Ôunpuu S Anand S Global burden of cardiovascular diseases. Part I: General considerations, the epidemiologic transition, risk factors, and impact of urbanization Circulation 2001 104 2746 2753 10.1161/hc4601.099487 11723030

[B5] Australian Institute of Health and Welfare Rheumatic heart disease: all but forgotten except among Aboriginal and Torres Strait Islander peoples. Bulletin no. 16. AIHW Cat. No. AUS 48 2004 Canberra: AIHW

[B6] Kaplan GA Keil JE Socioeconomic factors and cardiovascular disease: a review of the literature Circulation 1993 88 1973 1998 8403348 10.1161/01.cir.88.4.1973

[B7] Australian Institute of Health and Welfare Socioeconomic inequalities in cardiovascular disease in Australia. Bulletin no. 37. AIHW Cat. No. AUS 74 2006 Canberra: AIHW

[B8] Turrell G Mathers C Socioeconomic inequalities in all-cause and specific-cause mortality in Australia: 1985-1987 and 1995-1997 Int J Epidemiol 2001 30 231 239 10.1093/ije/30.2.231 11369721

[B9] Cunningham J O'Dea K Dunbar T Weeramanthri T Shaw J Zimmet P Socioeconomic status and diabetes among urban Indigenous adults aged 15-64 years in the DRUID study Ethn Health 2008 13 23 37 10.1080/13557850701803130 18066736

[B10] Cunningham J Socioeconomic gradients in self-reported diabetes for Indigenous and non-Indigenous Australians aged 18-64 Aust N Z J Public Health 2010 34 S18 S24 10.1111/j.1753-6405.2010.00547.x 20618286

[B11] Cass A Cunningham J Snelling P Wang Z Hoy W End-stage renal disease in Indigenous Australians: a disease of disadvantage Ethn Dis 2002 12 373 378 12148708

[B12] Cass A Cunningham J Wang Z Hoy W Regional variation in the incidence of end-stage renal disease in Indigenous Australians Med J Aust 2001 175 24 27 11476198 10.5694/j.1326-5377.2001.tb143507.x

[B13] Cunningham J Socioeconomic status and self-reported asthma in Indigenous and non-Indigenous Australian adults aged 18-64 years: analysis of national survey data Int J Equity Health 2010 9 18 10.1186/1475-9276-9-18 20698967 PMC2928229

[B14] Braveman PA Cubbin C Egerter S Chideya S Marchi KS Metzler M Posner S Socioeconomic status in health research: one size does not fit all JAMA 2005 294 2879 2888 10.1001/jama.294.22.2879 16352796

[B15] Williams DR Mohammed SA Leavell J Collins C Race, socioeconomic status and health: complexities, ongoing challenges, and research opportunities Ann N Y Acad Sci 2010 1186 69 101 10.1111/j.1749-6632.2009.05339.x 20201869 PMC3442603

[B16] Australian Bureau of Statistics National Health Survey and National Aboriginal and Torres Strait Islander Health Survey: Data Reference Package, 2004-05. ABS Cat. No. 4363.0.55.001 2006 Canberra: Australian Bureau of Statistics

[B17] Australian Bureau of Statistics National Aboriginal and Torres Strait Islander Health Survey: Expanded Confidentialised Unit Record File, Technical Manual, 2004-05. ABS Cat. No. 4715.0.55.002 2006 Canberra: Australian Bureau of Statistics

[B18] Australian Bureau of Statistics National Aboriginal and Torres Strait Islander Health Survey: Users' Guide, 2004-05. ABS Cat. No. 4715.0.55.004 2006 Canberra: Australian Bureau of Statistics

[B19] Australian Bureau of Statistics National Health Survey 2004-05. Summary of Results. ABS Cat. No. 4364.0 2006 Canberra: Australian Bureau of Statistics

[B20] Australian Bureau of Statistics National Aboriginal and Torres Strait Islander Health Survey 2004-05. ABS Cat. No. 4715.0 2006 Canberra: Australian Bureau of Statistics

[B21] Australian Bureau of Statistics National Health Survey: Users' Guide - Electronic Publication, Australia, 2004-05. ABS Cat. No. 4363.0.55.001 2006 Canberra: Australian Bureau of Statistics

[B22] Australian Bureau of Statistics Remote Access Data Laboratory (RADL) User Guide. ABS Cat. No. 1406.0.55.022, Version 4, March 2006 2006 Canberra: Australian Bureau of Statistics

[B23] Donath SM How to calculate standard errors for population estimates based on Australian National Health Survey data Aust N Z J Public Health 2005 29 565 571 10.1111/j.1467-842X.2005.tb00252.x 16366069

[B24] Paradies Y A systematic review of empirical research on self-reported racism and health Int J Epidemiol 2006 35 888 901 10.1093/ije/dyl056 16585055

[B25] Harris R Tobias M Jeffreys M Waldegrave K Karlsen S Nazroo J Effects of self-reported racial discrimination and deprivation on Māori health and inequalities in New Zealand: cross-sectional study Lancet 2006 367 2005 2009 10.1016/S0140-6736(06)68890-9 16782491

[B26] Harris R Tobias M Jeffreys M Waldegrave K Karlsen S Nazroo J Racism and health: the relationship between experience of racial discrimination and health in New Zealand Soc Sci Med 2006 63 1428 1441 10.1016/j.socscimed.2006.04.009 16740349

[B27] Rosmund R Role of stress in the pathogenesis of the metabolic syndrome Psychoneuroendocrinology 2005 30 1 10 10.1016/j.psyneuen.2004.05.007 15358437

[B28] Black PH The inflammatory response is an integral part of the stress response: implications for atherosclerosis, insulin resistance, type II diabetes and metabolic syndrome X Brain Behav Immun 2003 17 350 364 10.1016/S0889-1591(03)00048-5 12946657

[B29] Human Rights and Equal Opportunities Commission 'Bringing Them Home' - Report of the National Inquiry into the Separation of Aboriginal and Torres Strait Islander Children from their Families 1997 Sydney: Human Rights and Equal Opportunities Commission

[B30] Brunner E Marmot M Marmot M, Wilkinson RG Social organization, stress and health Social Determinants of Health 1999 Oxford (UK): Oxford University Press 17 43

[B31] LaVeist TA Thorpe RJ Galarraga JE Bower KM Gary-Webb TL Environmental and socio-economic factors as contributors to racial disparities in diabetes prevalence J Gen Intern Med 2009 24 1144 1148 10.1007/s11606-009-1085-7 19685264 PMC2762509

[B32] Paradies YC Montoya MJ Fullerton SM Racialized genetics and the study of complex diseases: the thrifty genotype revisited Perspect Biol Med 2007 50 203 227 10.1353/pbm.2007.0020 17468539

[B33] Kagawa Y Yanagisawa Y Hasegawa K Suzuki H Yasuda K Kudo H Single nucleotide polymorphisms of thrifty genes for energy metabolism: evolutionary origins and prospects for intervention to prevent obesity-related diseases Biochem Biophys Res Commun 2002 295 207 222 10.1016/S0006-291X(02)00680-0 12150934

[B34] Krieger N Williams DR Moss NE Measuring social class in US public health research Annu Rev Public Health 1997 18 341 378 10.1146/annurev.publhealth.18.1.341 9143723

[B35] Lynch J Kaplan G Berkman LF, Kawachi I Socioeconomic position Social Epidemiology 2000 New York: Oxford University Press 13 35

[B36] Kennedy B Firman D Indigenous SEIFA - revealing the ecological fallacy 2004 Paper presented at: Population and Society: Issues, Research, Policy. 12th Biennial Conference of the Australian Population Association; Canberra, Australia

[B37] Daly AE Smith DE Indigenous Household Demography and Socioeconomic Status: The Policy Implications of the 1996 Census Data. CAEPR Discussion Paper No. 181/1999 1999 Canberra: Centre for Aboriginal Economic Policy Research

[B38] Karlamangla AS Stein Merkin S Crimmins EM Seeman TE Socioeconomic and ethnic disparities in cardiovascular risk in the United States, 2001-2006 Ann Epidemiol 2010 20 617 628 10.1016/j.annepidem.2010.05.003 20609342 PMC2901883

[B39] Lutsey PL Diez Roux AV Jacobs DR Jr Burke GL Harman J Shea S Folsom AR Associations of acculturation and socioeconomic status with subclinical cardiovascular disease in the Multi-Ethnic Study of Atherosclerosis Am J Public Health 2008 98 1963 1970 10.2105/AJPH.2007.123844 18511718 PMC2575668

[B40] Cox AM McKevitt C Rudd AG Wolfe CDA Socioeconomic status and stroke Lancet Neurol 2006 5 181 188 10.1016/S1474-4422(06)70351-9 16426994

[B41] Grotto I Huerta M Sharabid Y Hypertension and socioeconomic status Curr Opin Cardiol 2008 23 335 339 10.1097/HCO.0b013e3283021c70 18520717

[B42] Thomas DP Briggs V Anderson IP Cunningham J The social determinants of being an Indigenous non-smoker Aust N Z J Public Health 2008 32 110 116 10.1111/j.1753-6405.2008.00185.x 18412679

